# Oligomannose-Rich Membranes of Dying Intestinal Epithelial Cells Promote Host Colonization by Adherent-Invasive *E. coli*

**DOI:** 10.3389/fmicb.2018.00742

**Published:** 2018-04-18

**Authors:** Tetiana Dumych, Nao Yamakawa, Adeline Sivignon, Estelle Garenaux, Stefania Robakiewicz, Bernadette Coddeville, Antonino Bongiovanni, Fabrice Bray, Nicolas Barnich, Sabine Szunerits, Christian Slomianny, Martin Herrmann, Sébastien G. Gouin, Alexander D. Lutsyk, Luis E. Munoz, Frank Lafont, Christian Rolando, Rostyslav Bilyy, Julie M. J. Bouckaert

**Affiliations:** ^1^Department of Histology, Cytology and Embryology, Danylo Halytsky Lviv National Medical University, Lviv, Ukraine; ^2^Unité de Glycobiologie Structurale et Fonctionnelle, UMR8576 Centre National de la Recherche Scientifique, University of Lille, Villeneuve d'Ascq, France; ^3^Université Clermont Auvergne, Institut National de la Santé et de la Recherche Médicale U1071, USC-INRA 2018, M2iSH, Clermont-Ferrand, France; ^4^Cellular Microbiology and Physics of Infection Group-Center of Infection and Immunity of Lille, Institut Pasteur de Lille, Centre National de la Recherche Scientifique UMR8204, INSERM U1019, Lille Regional Hospital University Centre, University of Lille, Lille, France; ^5^Miniaturisation pour l'Analyse, la Synthèse et la Protéomique, USR 3290 Centre National de la Recherche Scientifique, University of Lille, Villeneuve d'Ascq, France; ^6^Institut Supérieur de l'Electronique et du Numérique, University of Lille, Centrale Lille, UMR 8520—IEMN, University Valenciennes, Lille, France; ^7^Laboratoire de Physiologie Cellulaire, Institut National de la Santé et de la Recherche Médicale U.1003, University of Lille, Villeneuve d'Ascq, France; ^8^Department of Internal Medicine 3—Rheumatology and Immunology, Friedrich-Alexander-University Erlangen-Nürnberg and Universitätsklinikum Erlangen, Erlangen, Germany; ^9^Chimie Et Interdisciplinarité, Synthèse, Analyse, Modélisation, UMR 6230 Centre National de la Recherche Scientifique, Université Nantes Angers Le Mans (L'UNAM), Nantes, France

**Keywords:** CEACAM6, adherent-invasive *E. coli*, apoptotic cell-derived membranous vesicles, oligomannose glycans, Crohn's disease

## Abstract

A novel mechanism is revealed by which clinical isolates of adherent-invasive *Escherichia coli* (AIEC) penetrate into the epithelial cell layer, replicate, and establish biofilms in Crohn's disease. AIEC uses the FimH fimbrial adhesin to bind to oligomannose glycans on the surface of host cells. Oligomannose glycans exposed on early apoptotic cells are the preferred binding targets of AIEC, so apoptotic cells serve as potential entry points for bacteria into the epithelial cell layer. Thereafter, the bacteria propagate laterally in the epithelial intercellular spaces. We demonstrate oligomannosylation at two distinct sites of a glycoprotein receptor for AIEC, carcinoembryonic antigen related cell adhesion molecule 6 (CEACAM6 or CD66c), on human intestinal epithelia. After bacterial binding, FimH interacts with CEACAM6, which then clusters. The presence of the highest-affinity epitope for FimH, oligomannose-5, on CEACAM6 is demonstrated using LC-MS/MS. As mannose-dependent infections are abundant, this mechanism might also be used by other adherent-invasive pathogens.

## Introduction

Microbes express an arsenal of virulence factors with which they highjack the normal functions of the host. A well-known example of those functions is cell-cell adhesion. The adaptive immune response of the host can interfere with the microbe's recognition and binding of cell adhesion molecules, but adaptive immune responses are slow to develop after initial exposure to a new pathogen. Specific clones of B and T cells have to be activated and expanded, and it can take up to a week before adaptive immune responses are effective. During the first hours and days following exposure to a pathogen, the innate immune system provides protection. Unlike adaptive immune responses, innate immune responses are not pathogen-specific, and rely notably on complement proteins to evoke signaling aimed at eliminating the invaders. Complement activation leads to disruption of the microbial membrane, which attracts phagocytes and leads to an inflammatory response. The phagocytic cells secrete degradative enzymes, antimicrobial peptides, and reactive oxygen species aimed at killing the invading microbes. Uropathogenic *E. coli* (UPEC), which are the causal agents of most urinary tract infections (UTIs), express virulence factors that allow them to colonize mammalian bladder cells (Stamm, 2002; Laupland et al., 2007). Adherent-invasive *E. coli* (AIEC) strains, which are associated with microbial dysbiosis in 37% of Crohn's disease (CD) patients (Darfeuille-Michaud et al., 1998, 2004), share a phylogenetic link with UPEC (Miquel et al., 2010). Both AIEC and UPEC strains can adhere to and interact with receptors on the epithelial linings of the gastrointestinal tract. They use the same protein surface appendages, namely, type 1 fimbriae with oligomannose-specific lectin FimH at their tips (Martinez et al., 2000; Kline et al., 2009), making them highly invasive. For the *fimH* gene, the prototypic *E. coli* strains LF82 and UTI89 are classified in the same phylogenetic group (Miquel et al., 2010; Conte et al., 2016) known as AIEC, which was used in this work.

Eukaryotic cells release vesicles under different conditions, such as apoptosis, stress, communication, and transportation (van der Pol et al., 2012). When superficial urothelial cells become infected with type-1 fimbriated, FimH-positive *E. coli*, the underlying cell layer undergoes accelerated cell proliferation and differentiation in response to bone morphogenetic protein 4 signaling (Mysorekar et al., 2009). Because of this efficient system of bacterial elimination through boosted epithelial renewal, invasive pathogens are more likely to infect patients with metabolic diseases associated with increased apoptosis, such as diabetes mellitus (Geerlings et al., 2002; Taganna et al., 2011). Apoptosis is a programmed and regulated physiological process of cell death that plays an essential role in tissue turnover. During apoptosis, cells shrink and compact themselves by directing superfluous materials into apoptotic blebs, named apoptotic cell-derived membranous vesicles (ACMV). Recently, we showed that apoptotic cells release two distinct types of ACMV, derived either from the endoplasmic reticulum (ER) or from the plasma membrane (PM) (Bilyy et al., 2012). ER-derived ACMV carrying immature glycoproteins are potentially important for FimH-mediated bacterial adhesion, analogous to the macrophage system where the oligomannosidic glycans exposed on apoptotic cells and ACMV serve as an antenna for macrophage recognition (Tomin et al., 2015).

The human adhesion molecule, carcinoembryonic antigen-related cell adhesion molecule 6 (CEACAM6, also known as CD66c or NCA) favors colonization by AIEC; its gene expression is upregulated by inflammatory cytokines and possibly by microbes (Chassaing et al., 2011). CEACAM6 is a complex, highly glycosylated protein that belongs to the large immunoglobulin (Ig) superfamily and consists of one IgV-like and two IgC2-like domains (Thompson et al., 1991). The *ceacam6* gene is overexpressed in most carcinomas, including those of the gastrointestinal, respiratory and genitourinary tracts (Gemei et al., 2013). Increased serum levels of CEACAM6 serve as prognostic indicators of chronic inflammation in CD patients, given that no CEACAM6 production and mannosylation were observed in healthy ileal mucosa (Barnich et al., 2007). In more than 35% of CD patients with ileal involvement, the abundance of mannosidic structures at the ileal mucosa is elevated due to overexpression of *ceacam6* by ileal epithelial cells, which favors AIEC colonization. In turn, AIEC colonization induces increased *ceacam6* expression, thus further facilitating colonization. Recently, a discrepancy between the predicted and observed molecular weights of CEACAM6 led to the identification of a glycosylation site at Asn-197 containing a paucimannosidic glycan (Thaysen-Andersen et al., 2015). In parallel, it has been reported that AIEC effectively binds oligomannose glycans (Brument et al., 2013). However, a deeper understanding of the interaction between host cells and AIEC is needed in order to combat AIEC infections. Here, we follow how AIEC interacts with oligomannosidic receptors upon infection of human intestinal Caco-2 cells and then spreads in epithelial cells to promote its survival, replication and biofilm formation (Oligomannose-rich membranes of dying cells promote host cell colonization, Graphical Abstract).

**Graphical Abstract:**
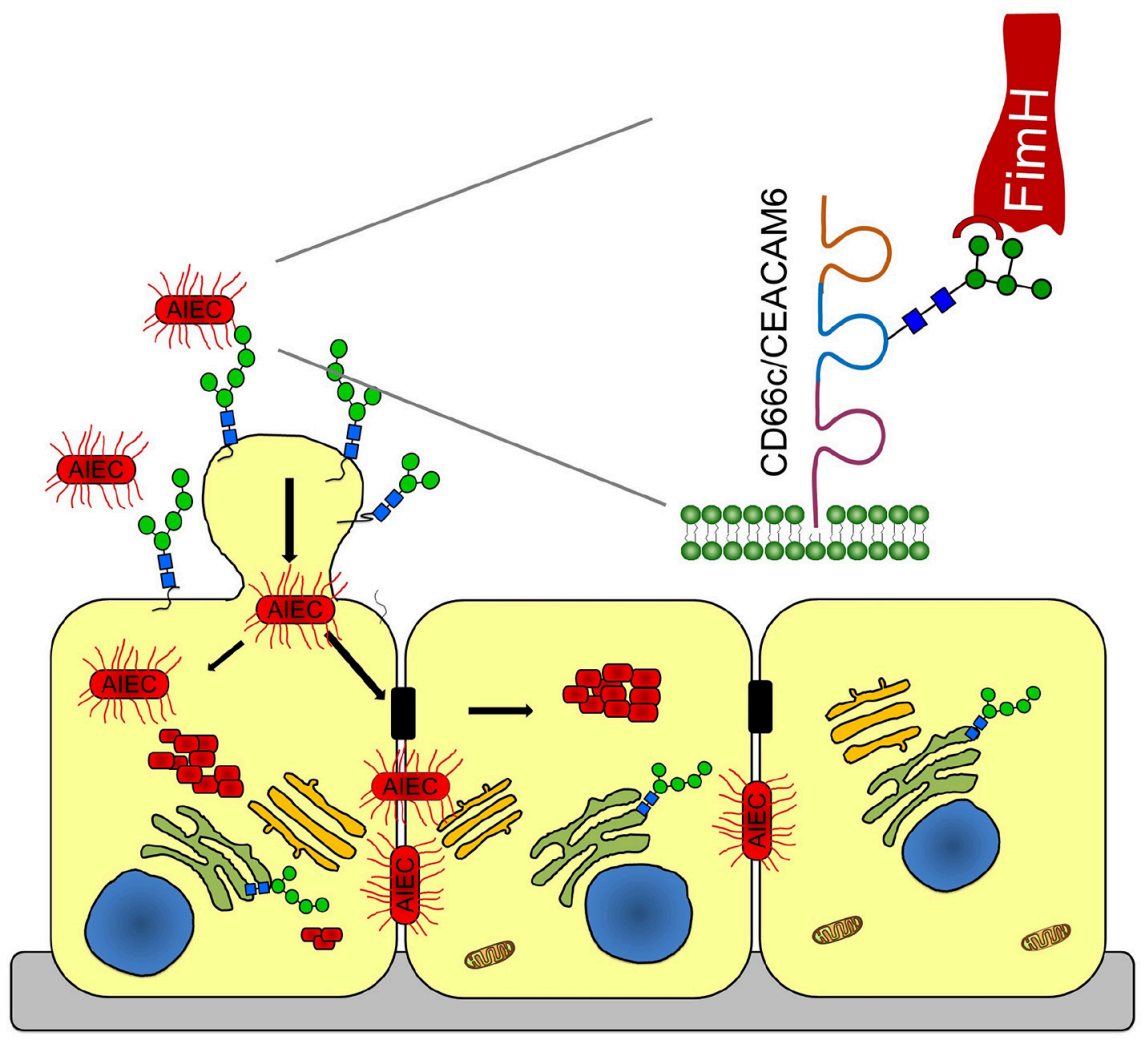
A Sweet Shuttle for Adherent-Invasive *Escherichia coli* Through Epithelial Membranes.

## Results

### Effect of *fimH*-expressing *E. coli* on the infection of human HeLa cells

**Figure 1:**
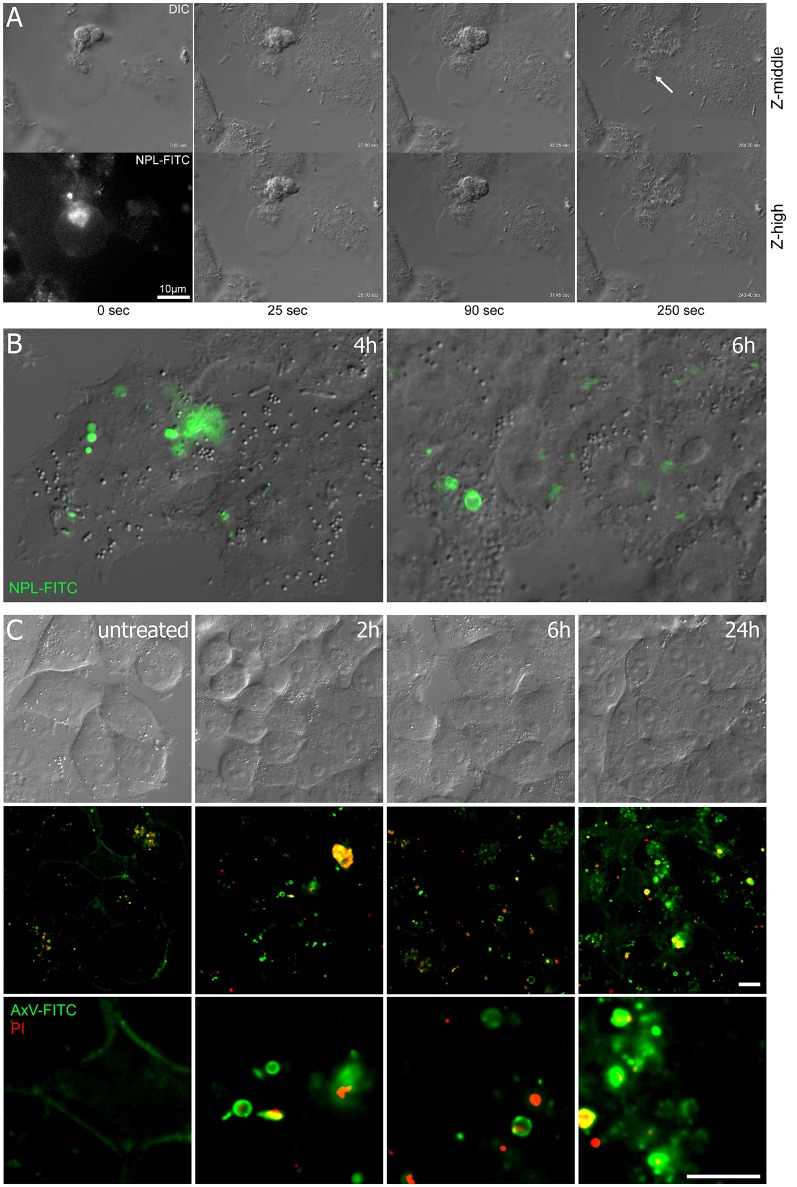
Infection of HeLa cells with AIEC induces surface blebbing. **(A)** Video microscopy over a time course of several minutes, where AIEC that had been co-incubated for 6 h with HeLa cells attach to and then penetrate (arrow) into membranous vesicles of blebbing cells. **(B)** Co-incubation of HeLa cells with *E. coli* UTI89 cells resulted in the appearance of oligomannose-rich blebs, as visualized using NPL-FITC lectin in DIC and fluorescence microscopy (merged images). **(C)** Bleb formation in HeLa cells treated with the FimH protein was observed as early as 2 h after infection. DIC imaging is shown in the upper row and fluorescence microscopy with annexin V-FITC (green) and PI (red) in the two lower rows. Scale bar = 10 μm.

The binding of *E. coli* to epithelial cells is mediated by the type-1 fimbrial adhesin FimH (Hultgren et al., 1991; Martinez et al., 2000). FimH is involved in the induction of apoptosis (Klumpp et al., 2006; Thumbikat et al., 2009) and we asked whether FimH-induced apoptosis coincides with the occurrence of oligomannosidic glycans. Bleb production was prominent in HeLa cells upon interaction with the type-1 fimbriated *E. coli* UTI89 strain. Differential interference contrast (DIC) microscopy allowed visualization of bacterial infection of ACMV (Figure 1A). Oligomannosidic glycans on the surface of ACMV were detected using the mannose-specific lectin, *Narcissus pseudonarcissus* (NPL), conjugated with fluorescein isothiocyanate (FITC) (Figure 1B). Since bacterial binding to oligomannosidic glycans is mediated by the FimH adhesin (Bouckaert et al., 2006), purified FimH lectin domain (10 μg/mL) was added to HeLa cells and cell viability was analyzed using annexin V-FITC/propidium iodide (PI) fluorescent staining. DIC images showed abundant bleb formation on the surfaces of the human epithelial cells as early as 2 h after treatment with the FimH lectin. The number of annexin V-positive blebs, which resembled apoptotic vesicles (ACMV), increased with time (Figure 1C).

**Figure 2:**
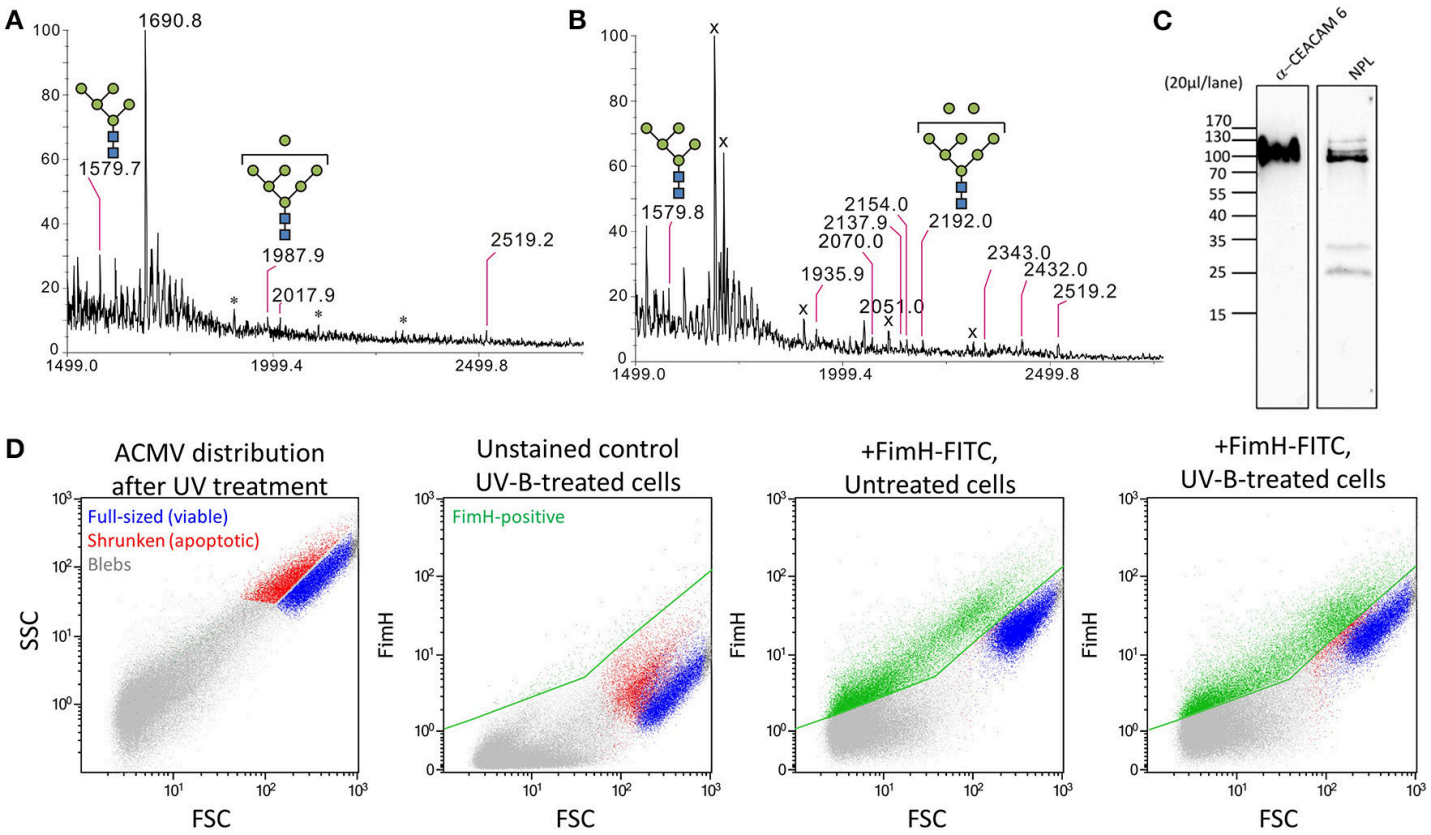
FimH binds to ACMV-derived oligomannoside glycans. **(A)** FimH-induced blebs possess oligomannose-glycans, which are known FimH-binding targets, resemble the blebs **(B)** seen during early apoptosis induced by UV-B irradiation. **(C)** Western blot of FimH-induced blebs shows CEACAM6 and oligomannosidic residues detected by using antibodies and *Narcissus pseudonarcissus* lectin (NPL), respectively. **(D)** Flow cytometry of FimH binding to Caco-2 cells and their ACMV. Both apoptotic cells and ACMV are effectively bound by FimH-FITC lectin (positive population is colored green).

MALDI-TOF MS/MS spectra (tandem mass spectrometry) were used to characterize *N*-glycans obtained from isolated membranous vesicles. HeLa cells or the colonic epithelial cells Caco-2 were exposed to FimH. UV-B irradiation served as a positive control for ACMV formation and no irradiation served as a negative control. As shown in the results obtained using Caco-2 cells, the membranous vesicles produced by the cells exposed to FimH possessed oligomannose-5 (m/z = 1579.7) and oligomannose-7 glycans (m/z = 1987.9) (Figure 2A), like the ACMV induced by UV-B irradiation [oligomannose-5 and oligomannose-8 glycans (m/z = 2192.0)] (Figure 2B). No oligomannosidic glycans were detected in the membranous vesicles (exosomes, blebs) produced by the untreated cells under normal circumstances. These results are similar to those obtained with HeLa cells. (Figure S1). Western blot analysis of the bleb fraction of FimH-treated Caco-2 cells revealed that the mobility of CEACAM6 was similar to that of the proteins detected by NPL binding (Figure 2C). Staining of Caco-2 cells with FimH-FITC lectin confirmed their binding to subcellular membranous microvesicles or to ACMV, as well as to subpopulations of full-sized or shrunken cells (Figure 2D). Interestingly, high levels of CEACAM6 were present on immortalized cells of intestinal epithelial origin (Caco-2, HT29) and those cells can be used to extract and purify CEACAM6 (Figure S2), whereas much lower levels were present on HeLa cells (Figure S3). CEACAM6 levels were low or absent on primary human cells such as lymphocytes and monocytes.

### AIEC infection relies on interaction of FimH with mannose and involves bacterial dissemination in the intercellular spaces

**Figure 3:**
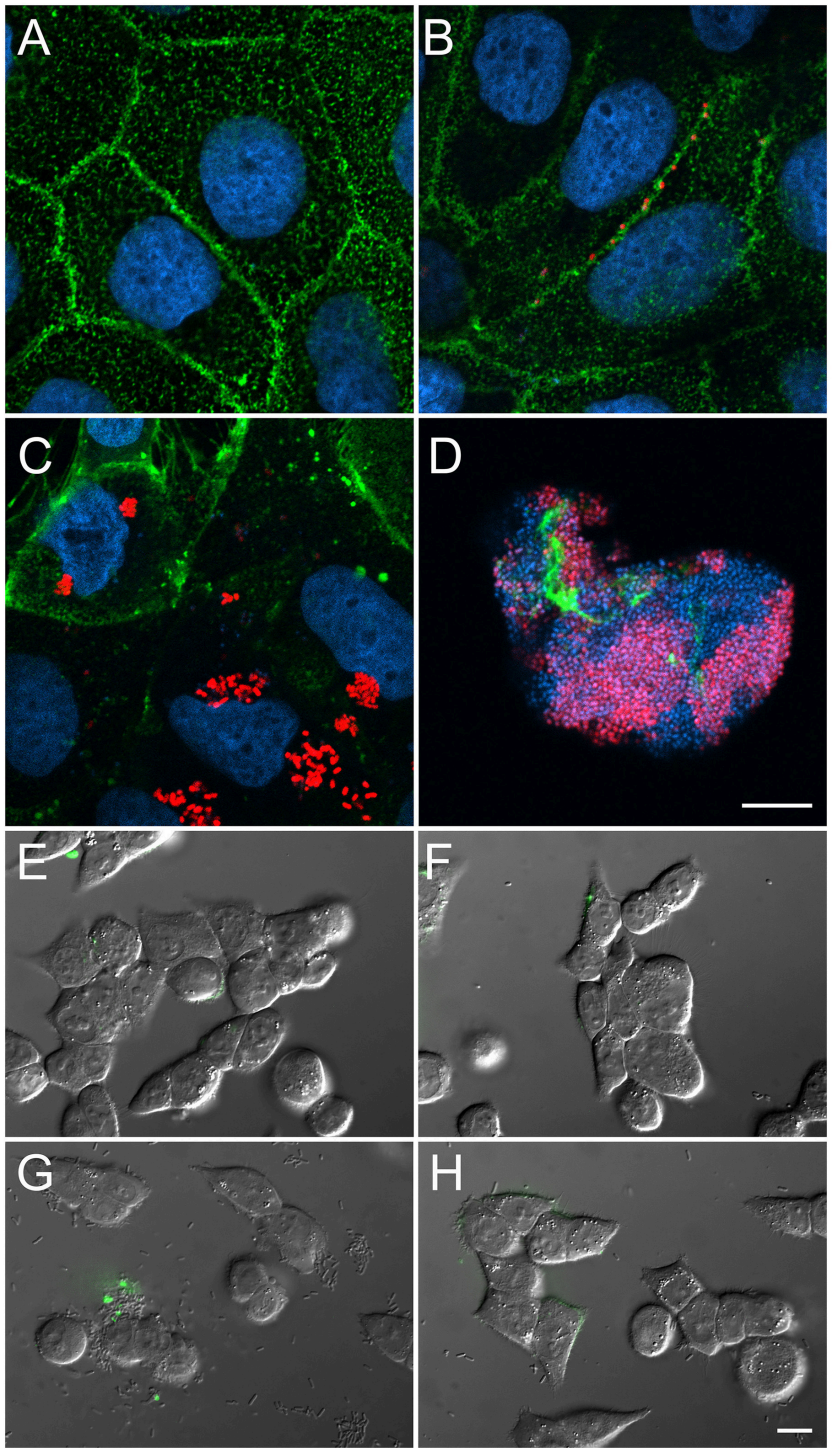
Bacterial colonization of Caco-2 cells requires surface exposure of the oligomannose. Bacteria penetrate through the intercellular spaces and then invade the cells. Their binding is dependent on FimH-oligomannose interactions. Nuclear DNA was visualized with DAPI staining (blue) and cell membranes were stained with fluorescent wheat germ agglutinin (WGA-FITC) (green, **A–D**). *E. coli* was transformed with a plasmid encoding NIR-fluorescent protein TurboFP635 (red, **A–D**). **(A)** Semi-confluent uninfected Caco-2 cells. **(B,C)** Caco-2 cells infected with *E. coli* LF82 for 5 h and treated with gentamicin for 19 h. **(D)** Infection for 24 h with LF82 without gentamicin treatment. **(E)** Untreated cells. **(F)** Infection with *E. coli* LF82-Δ*fimH*. **(G)** Wild-type *E.coli* LF82 and **(H)** *E.coli* LF82 co-incubated with 500 nM of a thiazolylamine mannose-based inhibitor. **(E–H)** NPL-FITC was used to visualize oligomannosidic glycans (green, overlayed). Scale bar = 10 μm.

Little is known about how AIEC infects epithelial cells. The AIEC LF82 strain, transformed with a plasmid coding for the near-infra red (NIR)-fluorescent protein TurboFP635, was co-incubated with Caco-2 cells for 5 h. Next, the cell co-culture was treated with gentamicin to kill extracellular bacteria, or left untreated (Figure 3A). Video lapse microscopy revealed that AIEC invaded Caco-2 cell within 4–5 h after infection. During the progression of infection, *E. coli* was observed near intercellular junctions (Figure 3B and Figure S4). Upon invasion, the bacteria multiplied intracellularly and spread rapidly (Figure 3C). At the later stages of infection, the bacteria formed biofilms (Figure 3D). In the actively growing biofilm, the nucleoids of all bacteria were prominently stained with the DNA-specific dye (DAPI), but only part of the population was already in a state to produce the TurboFP635 NIR-fluorescent protein. Similar to these results, time-lapse confocal microscopy on healthy Caco-2 cells (Figure 3E), performed without arresting extracellular bacterial growth, revealed the formation of intracellular bacterial communities and their propagation inside the intercellular spaces 6–19 h after initial infection (Figure 3G and Figure S4). Bacterial binding and invasion and oligomannose exposure were abrogated in the non-piliated LF82-Δ*fimH* mutant (Figure 3F) and were diminished upon treatment with 500 nM of the potent FimH lectin inhibitor, 2-(α-d-mannopyranos-1-yl) amino-5-(4-methyl-2-(pyrazin-2-yl)thiazole-5-carbonyl)thiazole (Brument et al., 2013; Figure 3H). This confirms that adhesion to and invasion of Caco-2 cells by LF82 bacteria depend on the interaction of FimH with mannose.

### AIEC tends to bind dying cells and ACMV rich in mannose

**Figure 4:**
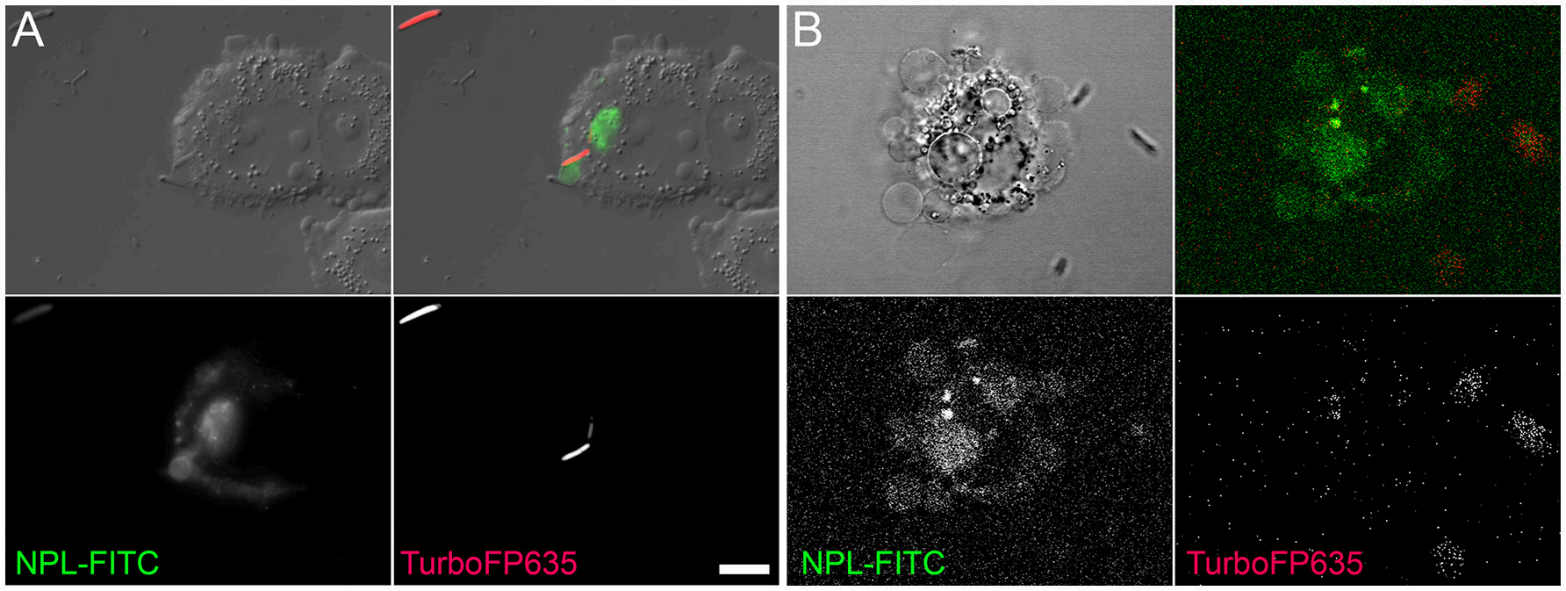
AIEC LF82 preferentially binds ACMV from dying cells due to their high mannose content. **(A)** Fluorescent video microscopy of HeLa cells after 7 h of co-culture with AIEC. **(B)** Confocal microscopy of live Caco-2 cells induced to apoptosis by UV-B irradiation. ACMV rich in mannose glycans were visualized by NPL-FITC. The bacteria produced NIR-fluorescent protein TurboFP635. Scale bar = 5 μm.

When AIEC LF82 was incubated with epithelial cells previously committed to apoptosis, they preferentially bound apoptotic bodies and ACMV rich in mannosidic glycans (Figure 4). In some cases, AIEC used ACMV to penetrate into dying cells (Figures 1A, 4B). These observations indicated that bacteria used dying (apoptotic) cells to penetrate the epithelial cell layer, and that after using the less protected cell surface for invasion, they go beyond the zone of tight junctions of the polarized cells to spread *via* the lateral membranes (Figure 3B).

### CEACAM6 mediates the interaction of the FimH adhesin with host cells

**Figure 5:**
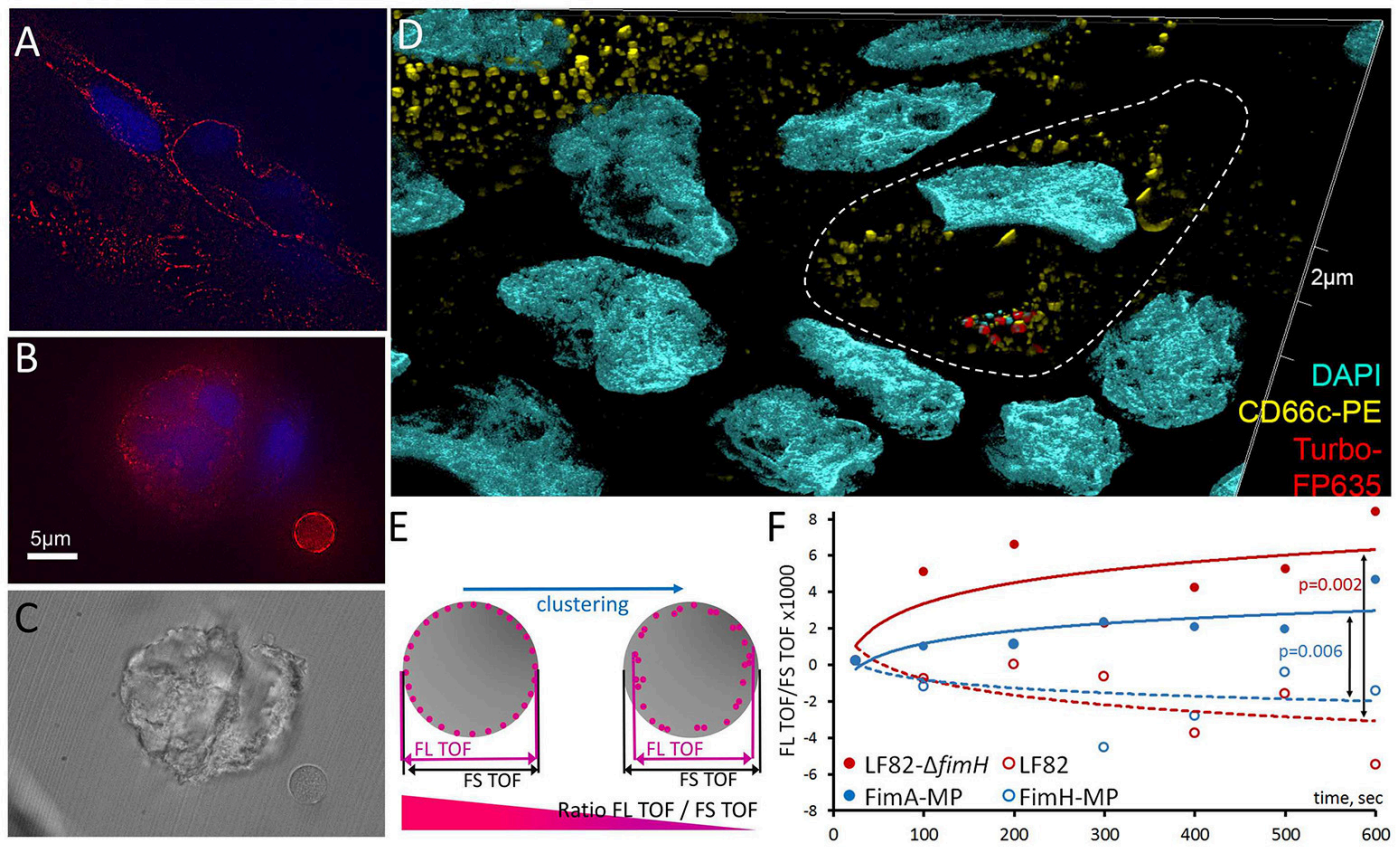
CEACAM6 is involved in the initial binding of AIEC. The CEACAM6 glycoprotein is produced and distributed in clusters **(A)** on the surface of viable cells and **(B,C)** on late apoptotic blebs or ACMV. **(D)** Structured illumination super-resolution microscopy of CEACAM6 clustering at the initial bacterial binding sites. **(E,F)** CEACAM6 forms clusters upon contact with the bacterial cells, or the FimH protein.

To identify the molecular targets of AIEC binding, we examined the production of the CEACAM6 protein, which is involved in initiating cell signaling upon bacterial invasion (Barnich et al., 2007; Chassaing et al., 2011). Using structured illumination microscopy (SIM), we found CEACAM6 distributed in clusters on the surfaces of viable cells, apoptotic cells and ACMV (Figures 5A–C). Examination of early bacterial infection events by SIM demonstrated the clustering of CEACAM6 at the places of initial bacterial binding (Figure 5D). To investigate the possible interaction of bacteria with CEACAM6, we measured the ratio of the time-of-flight (TOF) of cells in fluorescence (FL) and forward scatter (FS) (Fürnrohr et al., 2015). Clustering and capping of cell surface receptors resulted in a decrease of the TOF_FL_/TOF_FS_ ratio (Figure 5E). We stained Caco-2 cells with an anti-CEACAM6-PE antibody and immediately incubated them with AIEC or its component (FimH lectin). The TOF_FL_/TOF_FS_ ratio was measured for 10 min at >15,000 events per minute. The cells were treated with one of the following: AIEC LF82, AIEC LF82-Δ*fimH* mutant (negative control), 1 μm polystyrene microparticles (MP) conjugated with FimH (FimH-MP) or 1 μm polystyrene MP conjugated with FimA (major fimbrial subunit, negative control) (FimA-MP). For AIEC LF82 and FimH-MP, a significant decrease in TOF_FL_/TOF_FS_ ratio was observed for AIEC LF82 vs. AIEC LF82-Δ*fimH* (*p* = 0.002), and for FimH-MP vs. FimA-MP (*p* = 0.006), indicating that FimH-bearing bacteria and microparticles caused CEACAM6 clustering (Figure 5F).

### Two oligomannosidic glycans at Asn-197 and Asn-224 of the middle domain of CEACAM6 as potential receptors of the FimH adhesin

**Figure 6:**
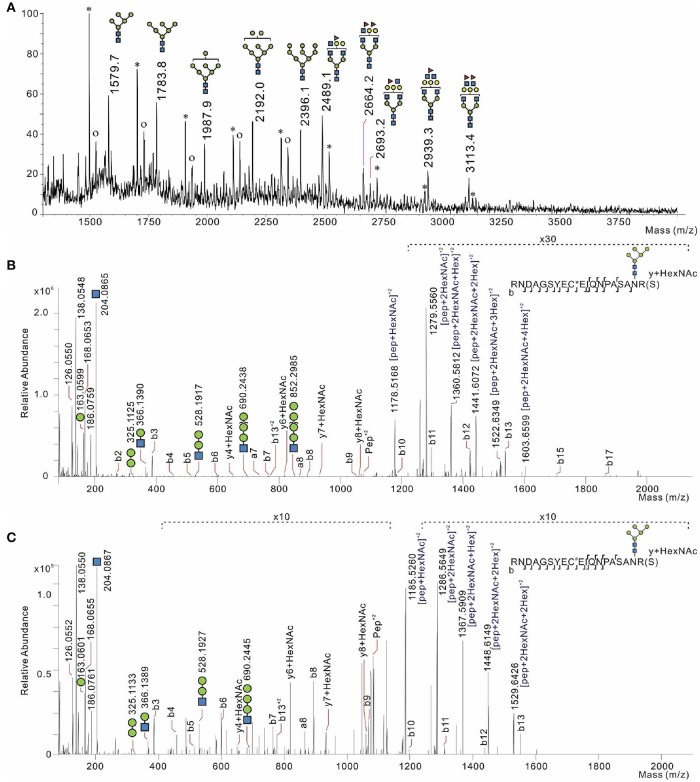
Glycosylation analysis of CEACAM6 identified two oligomannose-bearing glycosylation sites serving as receptors for the bacterial adhesin FimH. **(A)** MALDI TOF/TOF *N*-glycosylation profile of CEACAM6 isolated from Caco-2 cells treated with IFN-γ. The asterisk indicates a polyhexose contamination. The symbol “o” means oxonium structures of each high mannose glycan structure. LC-MS/MS profiles of the glycopeptide either **(B)** from recombinant human CEACAM6 protein or **(C)** from CEACAM6 isolated from intestinal epithelial cells after exposure to AIEC LF82 bacteria. Peptides were found with cysteine modification, **(B)** carbamidomethyl or **(C)** propionamide. Typical *N*-glycosylation (Man5GlcNAc2) was detected. Infection was for 3 h at a multiplicity of infection of 100 bacteria/cell.

Since bacteria rely on mannose-based interaction with surface targets on host cells (Barnich et al., 2007), we examined the glycosylation of CEACAM6. First, MALDI TOF/TOF MS analysis was performed on permethylated *N*-linked glycans of CEACAM6 isolated from IFNγ-stimulated Caco-2 cells (mimicking inflammatory conditions). We confirmed the presence of distinct oligomannose glycans in CEACAM6, including oligomannose-5 possessing the highest affinity for FimH (Figure 6A). The dissociation constant (*K*_*d*_) for oligomannose-3 and oligomannose-5 is about 20 nM, 10-fold higher than the affinity of FimH for oligomannose-6, -7, -8, and -9. The presence of Man5GlcNAc2-Asn *N*-glycosylation thus enables the FimH type-1 fimbrial adhesin to bind with the highest affinity (Bouckaert et al., 2006).

Subsequently, we infected intestinal epithelial cells with *E. coli* strains LF82 and K12-C600 in order to directly analyze the glycopeptides of CEACAM6. The typical LC-MS spectra of glycopeptides and its MS/MS fragmentation are shown in Figures 6B,C. From the obtained higher-energy collisional dissociation (HCD) fragmentation data, the proteome targeted against the CEACAM6 protein and the *N*-glycome were performed directly using Byonic™ v2.7.7 software. CEACAM6 had the highest score among all samples (Table S1). All Byonic™-annotated CEACAM6 glycopeptide-containing HCD spectra were inspected manually to verify the annotation.

In CEACAM6 recovered from both non-infected cells and cells infected with *E. coli* K12 or with *E. coli* LF82, two glycopeptides were detected despite low protein coverages: LQLSNGNMTLTLLSVK (191-206) and RNDAGSYECEIQNPASANR (207-225) (Table S1). HCD fragmentation did not detect these peptides in their non-glycosylated form, which means that they are frequently glycosylated. Indeed, the *N*-glycosylation sites on each peptide (Asn-197 and Asn-224) have been reported (Ramachandran et al., 2006; Chen et al., 2009).

Next we characterized the detected high-mannose type *N*-glycosylations on both asparagines (Table 1, Figures 6B,C). Interestingly, we have identified Man5GlcNAc2-Asn (Man5) on one of CEACAM6 glycopeptides, being a nanomolar-range carbohydrate ligand for FimH adhesins of type-1 fimbriated *E. coli* (Wellens et al., 2008).

**Table 1:** Presence of high mannose glycans on three different peptides.

**Sample condition name of data**	**Non-glycosylated peptide**		**Glycosylation**	**Glycopeptide**		**Modification**
	**Rt (min)**	**Scan**		**Rt (min)**	**Scan**	
**PEPTIDE SEQUENCE—RNDAGSYEC(PROPIONAMIDE/CARBAMIDOMETHYL)EIQNPASANR**						
*inGel control* CTRL_CEACAM6	–	–	Man5	32.909	6445	C (carbamidomethyl)
			Man6	32.851	6434	C (propionamide)
			Man7	32.934	6449	C (propionamide)
*inGel LF82* LF82_CEACAM6	–	–	Man5	33.219	6620	C (carbamidomethyl)
			Man6	32.955	6572	C (carbamidomethyl)
			Man7	32.950	6571	C (propionamide)
*inGel K12* K12_CEACAM6	–	–	Man5	33.220	6538	C (propionamide)
			Man6	32.809	6463	C (carbamidomethyl)
*eFASP recombinant* rh_CEACAM6	40.283	7041	Man5	38.217	6709	C (carbamidomethyl)
			Man6	37.915	6661	C (carbamidomethyl)
			Man7	37.561	6605	C (carbamidomethyl)
			Man8	37.391	6577	C (carbamidomethyl)
			Man9	37.379	6575	C (carbamidomethyl)
**PEPTIDE SEQUENCE—SDLVNEEATGQFHVYPELPKPSISSNNSNPVEDK**						
*eFASP recombinant* rh_CEACAM6	64.012	10872	Man5	61.786	10515	
			Man7	61.486	10467	
PEPTIDE SEQUENCE–LQ*LSN*GN*MTLTLLSVKR *POTENTIAL DEAMIDATION SITE						
*inGel control* CTRL_CEACAM6	–	–	Man6	67.165	12836	M (oxi) 1(deami)
			Man7	67.044	12812	M (oxi) 1(deami)
			Man8	66.628	12732	M (oxi) 2(deami)
*inGel LF82* LF82_CEACAM6	–	–	Man6	67.461	12954	M (oxi) 1(deami)
			Man7	64.396	12376	M (oxi) 1(deami)
			Man8	74.040	14199	2(deami)
*inGel K12* K12_CEACAM6	–	–	Man6	67.465	12835	M (oxi) 1(deami)
			Man7	67.111	12768	M (oxi) 1(deami)
			Man8	66.476	12642	M (oxi) 2(deami)
*eFASP recombinant* rh_CEACAM6	71.017	11999	Man5	57.172	9769	M(oxi) 1(deami)
			Man6	57.188	9771	M(oxi) 1(deami)
			Man7	57.697	9853	M(oxi)
			Man8	63.419	10776	

*inGel, in-gel trypsin digestion; control, CEACAM6 isolated from non-infected cells; LF82, CEACAM6 isolated from LF82-infected cells; K12, CEACAM6 isolated from K12-infected cells; recombinant, recombinant human CEACAM6; eFASP, enhanced filter-aided sample preparation; Rt, retention time; Scan, scan number of MS/MS spectrum; Man, mannose; M, modification; oxi, oxidation; deami, deamidation (1 or 2 times on this peptide)*.

Recombinant human CEACAM6 (rhCEACAM6) was chosen as a positive control sample of the CEACAM6 glycoprotein. By using enhanced filter-aided sample preparation (eFASP) on 50 μg of rhCEACAM6, a sequence coverage of 90% was identified. Abundant glycosylation on several peptides was characterized, not only on the two peptides we discovered, LQLSNGNMTLTLLSVK (191-206) and RNDAGSYECEIQNPASANR (207-225), but also on other peptides: ETIYPNASLLIQNVTQNDTGFYTLQVIK (99-126), SDLVNEEATGQFHVYPELPKPSISSNNSNPVEDK (127-160), and ANYRPGENLNLSC(Carbamidomethyl)HAASNPPAQYSWFINGTFQQSTQELFIPNITVNNSGSYMC(Carbamidomethyl)QAHNSATGLNR (247-310). Interestingly, in LQLSNGNMTLTLLSVK (191-206), RNDAGSYECEIQNPASANR (207-225) and SDLVNEEATGQFHVYPELPKPSISSNNSNPVEDK (127-160), we identified high-mannose type glycans (Man5, Man6, Man7, Man8, and Man9) (Table 1), together with complex types of glycosylation (Figure S5). Again, in the latter three peptides (191-206, 207-225, 127-160), over half of the peptides identified by HCD fragmentation were decorated with glycans, and in only one instance a non-glycosylated peptide was identified for the peptide LQLSNGNMTLTLLSVK (191-206). In the peptide ETIYPNASLLIQNVTQNDTGFYTLQVIK (99-126), we observed several types of glycan modifications, including the complex type *N*-glycosylation and *O*-glycosylation. As shown in Figure S5, two different complex type *N*-glycosylations were found on this peptide, but no high-mannose type *N*-glycosylation. Although we observed many traces of glycan fragmentation on the peptide (ANYRPGENLNLSC(carbamidomethyl)HAASNPPAQYSWFINGTFQQSTQELFIPNITVNNSGSYMC(carbamidomethyl)QAHNSATGLNR) (247-310), the peptide was too large (m/z = 7158) for complete characterization of its glycosylation.

Altogether, the results imply that the peptides we analyzed might carry both *N*-linked and *O*-linked glycosylation. Complete profiling of the *N*-glycosylated peptides might require further digestion and electron-transfer dissociation (ETD) fragmentation analyses. Infection of intestinal epithelial cells with non-pathogenic *E. coli* K12-C600 or with the LF82 bacterial strain, followed by affinity isolation of CEACAM6, its fragmentation, and LC-MS/MS analysis demonstrated that bacterial interaction with host cells did not lead to modification of the protein sequence of CEACAM6. As shown in Figure S6, the experimental and theoretical isotopic patterns obtained for the glycopeptide LQLSNGNMTLTLLSVKR with *N*-glycosylation HexNAc(2)Hex(7) at m/z = 1143.5365, z = 3 (C_139_H_245_N_26_O_7_0S_1_) overlap perfectly (−0.58 ppm of error), confirming the identification of the glycopeptide within the high accuracy and resolution of the Orbitrap Q-Exactive Plus mass spectrometer.

## Discussion

We propose that mannosidic glycans exposed on dying cells and on ACMV serve as an initial binding site for the AIEC LF82 lectin FimH. This proposal is based on the following: (1) our finding that dying cells expose binding targets for bacterial adhesins, (2) reports showing that multivalent binding of bacteria is required for host cell colonization (Weeks and Bouckaert, 2014; Yan et al., 2015) and (3) our data on the kinetics of AIEC localization and spreading. This FimH binding to mannosidic glycans is followed by recognition of CEACAM6 on the surface of intestinal epithelial cells in *cis* or *trans*. After initial binding by FimH, CEACAM6 clusters toward highly avid targets. A multivalent display of FimH, by covalently linking the FimCH chaperone-adhesin complex to microbeads, has been demonstrated previously as a requirement for its uptake into bladder cells (Martinez et al., 2000). After AIEC colonizes dying cells, which serve as an entry point into the epithelial layer, they move laterally through the membranes and colonize other neighboring cells. Initial binding of AIEC to cells might allow bacterial enzymes to cleave highly mannosylated glycans of CEACAM6 to produce an oligomannose-5 glycan that has the highest affinity for FimH. This could occur in *cis* or in *trans*. We previously reported *trans* cleavage by dying cells that desialylated their neighbors (Shkandina et al., 2012).

For UPEC, FimH first contacts bladder cells through uroplakin Ia (UPIa), which may induce apoptotic cell death mediated by inappropriate UPIIIa signaling (Zhou et al., 2001; Thumbikat et al., 2009). UPIa contains exclusively high-mannosylated *N*-glycans with 6, 7, 8, and 9 terminally exposed mannose residues (Xie et al., 2006). It has also been reported that UPEC can bind Gal(β1-3)GalNac epitopes during chronic cystitis (Conover et al., 2016). Nevertheless, the identity of the receptors involved in the binding and penetration of AIEC into the host cell has not been fully explored yet. During AIEC infections and upon IFN-γ pro-inflammatory stimulation, *ceacam6* expression is elevated in Caco-2 intestinal epithelial cells (Barnich et al., 2007). However, whether and how bacteria use this process for binding and invasion is unknown.

In CD patients, the intercellular space is more permeable and the regulation of tight junction proteins is disrupted (Turner, 2006). AIEC might exploit this weakness of the intestinal barrier for survival during CD manifestation, for otherwise they would be removed from the gut. Earlier (Brument et al., 2013), we reported that incubation of FimH with human Caco-2 cells led to only a moderate increase in the amount of apoptotic cells (~5%), which later underwent post-apoptotic, secondary necrosis. The same effect was observed in the current study when AIEC LF82 was incubated with human Caco-2 cells: after 5 h of co-infection followed by 19 h of co-incubation with gentamicin, most cells displayed bound bacteria, but only ~5% of them became apoptotic (by AxV staining) or necrotic (by DRAQ7 permeability). This phenomenon has important biological significance: AIEC uses live epithelial cells expressing mannosylated CEACAM6 for initial colonization. In parallel, a few dying cells might serve as an “entry point” into the epithelial cell bilayer when oligomannosidic glycans are exposed during apoptosis. Here, we report that bacteria can move between adjacent cells, and we propose that this lateral mobility increases their invasive capacity.

Colonization and invasion by bacteria usually require contact with an appropriate receptor. CEACAM6 is known to bind AIEC and is involved in the initiation of intracellular signaling (Chassaing et al., 2011). Strong CEACAM6 production has been observed in CD patients (Barnich et al., 2007). It is overexpressed in various cancers (Farina et al., 2009; Riley et al., 2009; Chen et al., 2010), and its silencing prevents tumor growth (Gemei et al., 2013). CEACAM6 has been proposed as a diagnostic marker of diseases (Farina et al., 2014; Sharma et al., 2017). Cells transfected with *ceacam6*-overexpressing vectors show constitutive production of proteins of the growth cycle and lack cellular polarization (Ilantzis et al., 2002).

The CEACAM6 protein has 12 potential *N*-glycosylation sites (Hefta et al., 1990), and the corresponding glycopeptides display distinct molecular weights. Glycosylation at Asn-197 and Asn-224 has been reported (Ramachandran et al., 2006; Chen et al., 2009). In azurophilic granules in pathogen-infected sputa from individuals with cystic fibrosis, Thaysen-Andersen and co-authors identified a paucimannosidic glycan on CEACAM6 with 2 GlcNAc and 2 Man, with or without Fuc (Thaysen-Andersen et al., 2015). In our study, we discovered the presence of high mannose type glycan on three peptides (Asn152, Asn197, and Asn224). Despite the limited amounts of CEACAM6 obtained from in-gel digestion, the presence of high-mannose type glycans on both Asn197 and Asn224 agrees with those found in rhCEACAM6. Notably, these three sites are located on the same C2-set Ig-like domain, which is immediately after the V-set Ig-like domain. Because we observed different types of glycosylation on other peptides of V-set and on the other C2-set Ig-like domain, it would be interesting to investigate and confirm the presence of high-mannose type glycan on the first C2-set domain. We found no significant difference between the glycan types in non-infected and infected samples (Table 1).

FimH binds specifically to oligomannosidic glycans, and synthetic analogs of naturally occurring mannosidic glycans were shown to effectively block bacterial cell adhesion (Almant et al., 2011; Alvarez Dorta et al., 2016; Chalopin et al., 2016). The glycosylation changes on the surface of cells that occur during various pathological conditions may serve as important susceptibility factors for urinary tract infection (Taganna et al., 2011). We show that FimH lectin from *E. coli* can induce not only apoptosis but also the formation of ER-derived blebs that exhibit immature high-mannose glycans. The discovery of previously unknown ER-derived blebs bearing oligomannose glycoepitopes (Bilyy et al., 2012) makes them potential targets for the binding of *E. coli* FimH adhesin. Blockage of oligomannose glycoepitopes or their elimination may be exploited to prevent invasion of host cells by pathogenic *E. coli*.

## Materials and methods

### Cell culture

Human cervical adenocarcinoma HeLa cells and human colorectal epithelial Caco-2 and T84 cells were obtained from the American Type Culture Collection (ATCC® CCL-2, HTB-37, CCL-248, respectively) and cultured in RPMI-1640 and Eagle's Minimum Essential Medium (EMEM), respectively (Sigma Chemical Co., USA). The media were supplemented with 4 mM L-glutamine, 10 mM HEPES buffer, 100 U/mL penicillin, 100 μg/mL streptomycin (PAA, Pasching, Germany), and fetal calf serum (Gibco-BRL, Eggenstein, Germany) at 20% (v/v) for Caco-2 cells and 10% for HeLa cells. Cultures were maintained under 5% CO_2_ at 37°C.

To detect surface exposure of phosphatidylserine and glycan, cells were stained with FITC-conjugated Annexin V (AxV-FITC, Böhringer, Mannheim, Germany) and lectins (Vector Laboratories). The cells were grown on slides of 22 × 22 mm, washed twice with Ringer's solution, and placed in 200 μL of reaction medium (Ringer's solution with 100 ng of AxV-FITC Nicoletti et al., 1991 or 1–5 μg/mL of lectins). The samples were analyzed immediately by fluorescence microscopy.

### Bacterial strains and growth

*E. coli* laboratory K12-C600, UPEC UTI89, and AIEC LF82 strains were grown overnight at 37°C in Luria-Bertani broth with vigorous aeration. *E. coli* K12-C600 was purchased from the American Type Culture Collection (ATCC 23724). UTI89 is a human cystitis isolate (Chen et al., 2006). The *E. coli* LF82 was isolated from a chronic ileal lesion of a Crohn's disease patient (Darfeuille-Michaud et al., 1998). For *in vitro* studies, bacteria were diluted 100-fold and allowed to enter into the exponential growth phase, after which they were harvested by centrifugation, washed twice in Dulbecco's phosphate-buffered saline (DPBS). Microbiology experiments at LNMU, U1071, and UGSF were carried out at biosafety level 2 according to regulations for working with clinical material. Appropriate personal protective equipment is worn, including lab coats and gloves. All procedures (cells cultures, bacterial infection of eukaryotic cells) that can cause infection from aerosols or splashes are performed within a biological safety cabinet. An autoclave is available for proper disposals. The laboratories have self-closing, lockable doors. A sink and eyewash station should be readily available. Biohazard warning signs are exposed when necessary. Outside personnel are restricted from entering when work is being conducted.

### Co-culture of epithelial cells with bacteria

Cells were grown in culture medium to 60% confluence. Before infection, cells were washed twice with DPBS and the growth medium was replaced by Hank's balanced salt solution (HBSS) containing 1 g/L of glucose. Bacteria were grown to an optical density of OD_600nm_ = 0.6–0.8. Epithelial cells were infected with 50–100 bacteria per cell in HBSS lacking antibiotics and incubated for 5 h at 37°C under 5% CO_2_. Next, they were washed three times with DPBS. Fresh HBSS containing 100 μg/mL of gentamicin was added to kill extracellular bacteria. After 1 h of incubation, host cells were washed twice with DPBS and either stained for microscopic analysis or incubated overnight with HBSS containing 15 μg/mL of gentamicin to assess bacterial infection. For microscopy, host cell membranes were stained using fluorescent wheat germ agglutinin (WGA-FITC) (Vector Laboratories) at a final concentration of 5 μg/mL, anti-CD66c-PE (phycoerythrin) (Affymetrix eBioscience) at a final concentration of 0.025 μg/mL, and 1 μM DAPI (Sigma-Aldrich).

### Induction of cell death and detection of apoptosis

Apoptotic cells were used to study bacterial FimH binding to ER-derived blebs. Apoptosis was induced by bacterial lectin FimH conjugated with carboxylated polystyrene microparticles (Polysciences, Inc., USA) or by UV-B irradiation at 180 mJ/cm^2^ for 90 s. FimH lectin was purified from *E. coli* as described (Wellens et al., 2008). Apoptosis rate was measured by flow cytometry, and by fluorescence microscopy by counting the AxV-positive cells using the ImageJ software.

### Flow cytometry

Flow cytometry was done using a Beckman Coulter Gallios flow cytometer according to the manufacturer's instructions. Data were analyzed with Kaluza software. CEACAM6 was detected using anti-human CD66C-PE (phycoerythrin) antibodies (Affymetrix eBiosciences) at a final concentration of 0.025 μg/mL. Necrotic cells were detected by propidium iodide (PI) permeability. Apoptotic cells were detected as AxV-FITC^+^/PI^−^ cells. Among other features, apoptosis is characterized by blebbing of the cellular membrane, leading to a decrease in the forward scatter (FSC) and an increase in the sideward scatter (SSC). To detect phosphatidylserine exposure on the cell surface, cells were stained with FITC-conjugated AxV in combination with PI (AxV/PI). 200,000 cells were stained for 30 min at 4°C with 200 ng of AxV-FITC and 500 ng PI in 500 μL Ringer's solution (Maueröder et al., 2016). The samples were analyzed immediately by flow cytometry.

### Construction of fluorescent bacterial strains

The construction of the plasmid *nadB::cat-kat* for the expression of the katushka (TurboFP635) near-infrared fluorescent protein was described previously (Yan et al., 2015). *E. coli* LF82 transformed with the plasmid was selected by overnight culture at 37°C on LB-agar supplemented with kanamycin and chloramphenicol. Bacterial colonies turned pink after four or more days of incubation at room temperature, confirming the production of TurboFP635 fluorescent protein.

### Microscopy

Fluorescence microscopy to observe DAPI, FITC-WGA, FITC-FimH, and FITC-AxV, PI, or the TurboFP635 protein expressed within *E. coli*, was done using a Zeiss AxioImager A1 Epifluorescence/DIC microscope equipped with an AxioCamMRm camera and corresponding fluorescence filters (all from Zeiss, Germany). Bright field signals were recorded with a conventional digital camera (Canon, Japan). In general, we performed 4-channel fluorescence microscopy (excitation maxima at 450, 515, 590, and 700 nm) and simultaneous DIC differential interference contrast and light microscopy. Confocal microscopy was performed using a Carl Zeiss LSM 780 microscope equipped with five lasers (405, 458, 488, 514, and 633 nm). Fluorescent labeling of lectins was done according to the general principles of lectin chemistry and bioconjugate techniques, as described (Bilyy et al., 2009).

### Structured illumination super-resolution microscopy

Structured illumination super-resolution microscopy (SIM) was performed using a Zeiss Axio Observer Z1 inverted microscope (Carl Zeiss, Germany) with a 63x/1.40 PLAN-APOCHROMAT objective. PALM-ELYRA PS1 lasers enable the excitation of probes at 405, 488, and 561 nm.

Camera 1002x1004 EMCCD (Andor Technology Ltd., UK) was used to acquire 15 SIM images (100 nm yx, 300 nm z) with five different phases for three different angular orientations of illumination for each SIM image). The images were processed with ZEN 2011 software (Carl Zeiss, Germany).

### Covalent coupling of proteins to carboxylated polystyrene microparticles

Covalent coupling of FimH and FimA lectin to Fluoresbrite® BB Carboxylate Microspheres 1.00 μm (Polysciences, Inc., USA, catalog No. 17458) was done according to the manufacturer's protocol (TDS 238C). We used 0.25 mL of 2.5% carboxylated microparticles. After covalent coupling of FimH or FimA, the microparticles were resuspended in 0.25 mL storage buffer [1x phosphate-buffered saline (PBS), 1% bovine serum albumin, 0.1% sodium azide, 5% glycerol)]. In total, 0.88 mg of FimH and 0.8 mg of FimA protein were conjugated with 1 mL of microparticles containing 4.55 x 10^10^ particles.

### Purification of CEACAM6

Human CEACAM6 protein was extracted from T84 cells overproducing CEACAM6 using a Pierce Direct IP Kit (Thermo Scientific, USA, catalog No. 26148) according to the manufacturer's protocol, with some modifications. Briefly, T84 were seeded in 150-cm^2^ at 2.5 × 10^7^ cells per Petri dish. Cells were infected for 3 h with K12-C600 or AIEC LF82 at a multiplicity of infection of 100 bacteria per cell. Cell monolayers were extensively washed with PBS and the cells were lysed with IP lysis buffer (Pierce Direct IP kit). CEACAM6 was extracted from total cell lysates by immunoprecipitation with antibodies against the human CEACAM6 (mouse monoclonal antibody to 9A6 to CEACAM6, Aldevron, catalog No. GM-0509) immobilized on an agarose support (Figure S2).

### Analysis of ACMV glycosylation by mass spectrometry

Apoptotic cell death of Caco-2 cells was induced by FimH lectin (10 FimH-MP per cell) or UV-B irradiation followed by 4 h incubation. The ACMV pellet was collected after centrifugation at 18,000 rpm (38,000 × g) for 60 min. Glycoproteins and glycolipids were separated by serial addition of chloroform/methanol in increasing ratios. Glycans from glycoproteins were released using 3 U of PNGaseF (1 U/μL, Roche) and purified on a 500-μL graphite column (Alltech). Samples were permethylated by adding dimethyl sulfoxide, sodium hydroxide, and iodomethane. Samples were washed with water and then dried under a stream of nitrogen. Before analysis by MALDI mass spectrometry, samples were diluted in methanol, and 0.5 μL was mixed with 0.5 μL of DHB matrix (2,5-dihydroxy benzoic acid in 50% acetonitrile and 0.1% trifluoroacetic acid solution).

### Enzymatic release of *N*-glycans and permethylation

The purified CEACAM6 extracted and purified from the Caco-2 cells was lyophilized and reconstituted in 4 mM dithiothreitol and 6 M guanidine hydrochloride in 50 mM Tris-HCl, pH 8. Reduction was carried out for 20 min at 100°C. After diluting the samples with 50 mM (NH_4_)HCO_3_ (ammonium bicarbonate) buffer, pH 8, they were digested overnight at 37°C with trypsin (1 μg per sample, G-Bioscience, Agro-Bio, La Ferte St Aubin, France). Trypsin was inactivated at −20°C. The digests were applied on a column packed with 20 mg of polymeric reversed phase Sepra™ ZT sorbent (Phenomenex). The samples were eluted with 5, 65, and 100% acetonitrile containing 0.1% trifluoroacetic acid. The peptide solution was dried and treated overnight with PNGaseF (1 U/μL, Roche).

Released *N*-glycans were recovered and purified using polymeric reversed phase Sepra™ ZT sorbent. *N*-glycans that were not retained by the stationary-phase medium were recovered in 0.1% trifluoroacetic acid solution. An aliquot of each sample was permethylated using the sodium hydroxide/dimethyl sulfoxide reagent (Ciucanu and Kerek, 1984). The permethylated derivatives were extracted in chloroform and repeatedly washed with water. Permethylation is a common derivatization method used in glycomics and can overcome the problems associated with ionization of glycans and loss of acidic structures. Permethylation stabilizes glycans and prevents loss of sialic acid during ionization.

### Analysis by MALDI-TOF-MS

Permethylated glycans were analyzed using a 4800+ Proteomics Analyzer MALDI-TOF/TOF (SCIEX, Framingham, MA, USA) in positive ion reflection mode with an accelerator voltage of 20 kV, a delay time of 200 ns, and grid voltage at 66%. The permethylated glycans were reconstituted in acetonitrile and 0.5 μL of each sample was spotted on the target plate and mixed with 0.5 μL of matrix DHB (2,5-dihydroxy benzoic acid in 50% acetonitrile and 0.1% trifluoroacetic acid solution).

### Trypsin enhanced filter-aided sample preparation (eFASP) digestion of recombinant human Ceacam6 (rHCEACAM6)

Ultrafilters from Amicon® (10 kDa cutoff limit; Millipore, Billerica, MA) were incubated overnight in 5% (v/v) Tween-20 (Sigma-Aldrich). Next, the filter units were rinsed thoroughly by three immersions in MS-grade water. eFASP digestion (Wiśniewski et al., 2009; Erde et al., 2014) was done on 50 μg of samples estimated from a Bradford assay. The rhCEACAM6 sample (CE6-H5223, AcroBiosystems) was mixed overnight at 4°C with the reducing buffer [(4% sodium dodecyl sulfate, 0.2% deoxycholic acid, 50 mM dithiothreitol, 200 mM (NH_4_)HCO_3_, pH 8.0] and then centrifuged at 13,000 × g for 15 min at 20°C. The filtrate was discarded, exchange buffer [8 M urea, 0.2% deoxycholic acid, 100 mM (NH_4_)HCO_3_, pH 8.0] was deposited on each filter unit, and centrifugation was resumed for 30 min. The reduced proteins were alkylated within the filter unit by adding alkylation buffer [8 M urea, 50 mM iodoacetamide and 100 mM (NH_4_)HCO_3_, pH 8.0] and incubating the unit at 37°C for 1 h with shaking in the dark. The alkylation buffer was exchanged three times with eFASP digestion buffer [50 mM (NH_4_)HCO_3_, 0.2% deoxycholic acid, pH 8.0]. Then, eFASP digestion buffer was added to each Amicon® unit, followed by 1 μg of trypsin (1:50 w/w). Digestion proceeded for 16 h at 37°C. To complete peptide recovery, the filters were rinsed twice with the 50 mM ammonium bicarbonate, pH 8.0, which was collected by centrifugation. Next, trifluoroacetic acid was added and quickly vortexed. Peptide precipitates were mixed with ethyl acetate and centrifuged at 13,000 g for 10 min. The organic supernatant was discarded and the step was repeated twice. Without disturbing the interface, as much of the upper organic layer as possible was removed and discarded. To remove residual ethyl acetate, the uncovered sample tube was placed in a thermomixer at 60°C in a fume hood for 5 min. Residual organic solvent and volatile salts were removed by vacuum drying in a SpeedVac. This step was repeated twice with 50% methanol.

### In-gel trypsin digestion of extracted CEACAM6

Cells were prepared under three conditions: no treatment of cells, and bacterial infection with K12 or LF82 for 3 h at a multiplicity of infection of 100. CEACAM6 attached to the beads of the affinity column was directly used for electrophoresis; the position of CEACAM6 on the gel was identified by comparison with the signal distribution in western blots. We used the anti-CEACAM6 antibody (9A6, Aldevron, Germany, catalog No. GM-0509) because CEACAM6 did not stain well with Coomassie blue (Figure S2). The targeted position of the gel was cut in small pieces and washed three times with 25 mM (NH_4_)HCO_3_ containing 50% (v/v) acetonitrile. Samples were dehydrated with acetonitrile and dried. The samples were reduced and alkylated by adding dithiotreitol and iodoacetamide, respectively. The gel pieces were washed with 25 mM (NH_4_)HCO_3_ containing 50% (v/v) acetonitrile, dehydrated with acetonitrile, and rehydrated in a digestion buffer containing 50 mM (NH_4_)HCO_3_ and 20 ng/μL of trypsin (Promega, sequencing grade). The digested products were extracted and dried completely by vacuum centrifugation.

### Analysis of *N*-glycans by liquid chromatography–tandem mass spectrometry (LC-MS/MS)

A nanoflow HPLC instrument (U3000 RSLC Thermo Fisher Scientific) was coupled on-line to a QExactive plus (Thermo Scientific) with a nanoelectrospray ion source. 1 μL of peptide mixture (corresponding to 200 ng of proteins) was loaded onto the pre-concentration trap (Thermo Scientific, Acclaim PepMap100C18, 5 μm, 300 μm i.d. × 5 mm) using partial loop injection for 5 min at a flow rate of 10 μL/min with buffer A (5% acetonitrile and 0.1% formic acid). The peptides were separated on a column (Acclaim PepMap100 C18, 2 μm, 75 mm i.d. × 500 mm) using a linear gradient of 5-50% buffer B (75% acetonitrile and 0.1% formic acid) at a flow rate of 250 nL/min at 45°C. The total time for a LC MS/MS run was about 120 min and each sample was injected three times.

### Data analysis: identification of glycosylation

The acquired raw files were analyzed with Byonic™ v2.7.7 (Protein Metrics Inc., San Carlos, CA, USA) to identify *N*-linked glycopeptides. The following parameters were used: CEACAM proteins of the Uniprot database (entry P40199 for human CEACAM6) (along with reversed proteins as decoys); tryptic peptides maximum three cleavages; mass accuracies within 15 ppm for both precursor and fragment ions; carbamidomethylation of cysteine was set as a fixed modification. The variable modifications considered were (i) oxidation of methionine and proline, (ii) deamidation of asparagine and glutamine, and (iii) phosphorylation of serine and threonine. For glycosylation, the database of “*N*-glycan human” including 57 *N*-glycan masses was selected. The acceptance criterion was a false discovery rate on the protein level below 1%. Peptide and glycan sequences were analyzed by ByonicTM from the HCD spectra and verified manually.

## Summary

Adherent-invasive *Escherichia coli* (AIEC) infects patients with ileal Crohn's disease. AIEC binds oligomannosidic cell surface receptors, penetrates into the cells, and can subsequently propagate within the epithelial layer. Whereas bacterial infections are correlated with an increased number of apoptotic cells, our results confirm that apoptotic cells may also serve as entry sites for pathogenic bacteria, from where they can spread to neighboring cells. Mass spectrometric characterization of carcinoembryonic antigen related cell adhesion molecule 6 (CEACAM6 or CD66c), a high-affinity receptor for the universal *E. coli* type-1 fimbrial adhesin, FimH, reveals oligomannosyl glycans at three different *N*-glycosylation sites. Unraveling the mechanism of the bacterial interaction with human intestinal epithelial cells will enable identification of compounds that specifically prevent AIEC adhesion without having a negative effect on other processes in the human body.

## Author contributions

TD, JB, and RB: designed the study, and cultured and infected the cells; NY and BC: performed the MALDI MS/MS experiments; AS, EG, and NB: developed the CEACAM6 extraction and purification protocol; TD, NY, FB, and CR: performed the LC-MS/MS and analyzed the data; TD, JB, AB, and FL: performed the super-resolution microscopy; CS: the confocal microscopy; RB, LM and MH: carried out the flow cytometric analysis of clustered cell surface receptors; SR: performed structural analysis; SG: synthesized the FimH-specific inhibitors; SS, MH and AL: assisted with and provided feedback on the study, including on the cell culture and infection co-culture model; TD, AS, NY, RB, and JB: wrote the manuscript.

### Conflict of interest statement

The authors declare that the research was conducted in the absence of any commercial or financial relationships that could be construed as a potential conflict of interest.
