# Supplementary material for: Oligomannose-Rich Membranes of Dying Intestinal Epithelial Cells Promote Host Colonization by Adherent-Invasive E. coli
Source: Front Microbiol. 2018 Apr 18;9:742. doi: 10.3389/fmicb.2018.00742 (PMC5915571; doi:10.3389/fmicb.2018.00742)
Supplement: Supplementary file 1 [file Presentation1.pdf]

Supplementary Material

Oligomannose-rich membranes of dying intestinal epithelial cells promote host colonization by adherent-invasive *E. coli*

Tetiana Dumych, Nao Yamakawa, Adeline Sivignon, Estelle Garenaux, Stefania Robakiewicz, Bernadette Coddeville, Antonino Bongiovanni, Fabrice Bray, Nicolas Barnich, Sabine Szunerits, Christian Slomianny, Martin Herrmann, Sébastien G. Gouin, Alexander D. Lutsyk, Luis Munoz, Frank Lafont, Christian Rolando, Rostyslav Bilyy*, Julie Bouckaert*

¶ These authors contributed equally to this work.
*** Correspondence:**
Rostyslav Bilyy r.bilyy@gmail.com,
Julie Bouckaert julie.bouckaert@univ-lille1.fr

1 Supplementary Figures and Tables

1.1 Supplementary Figures

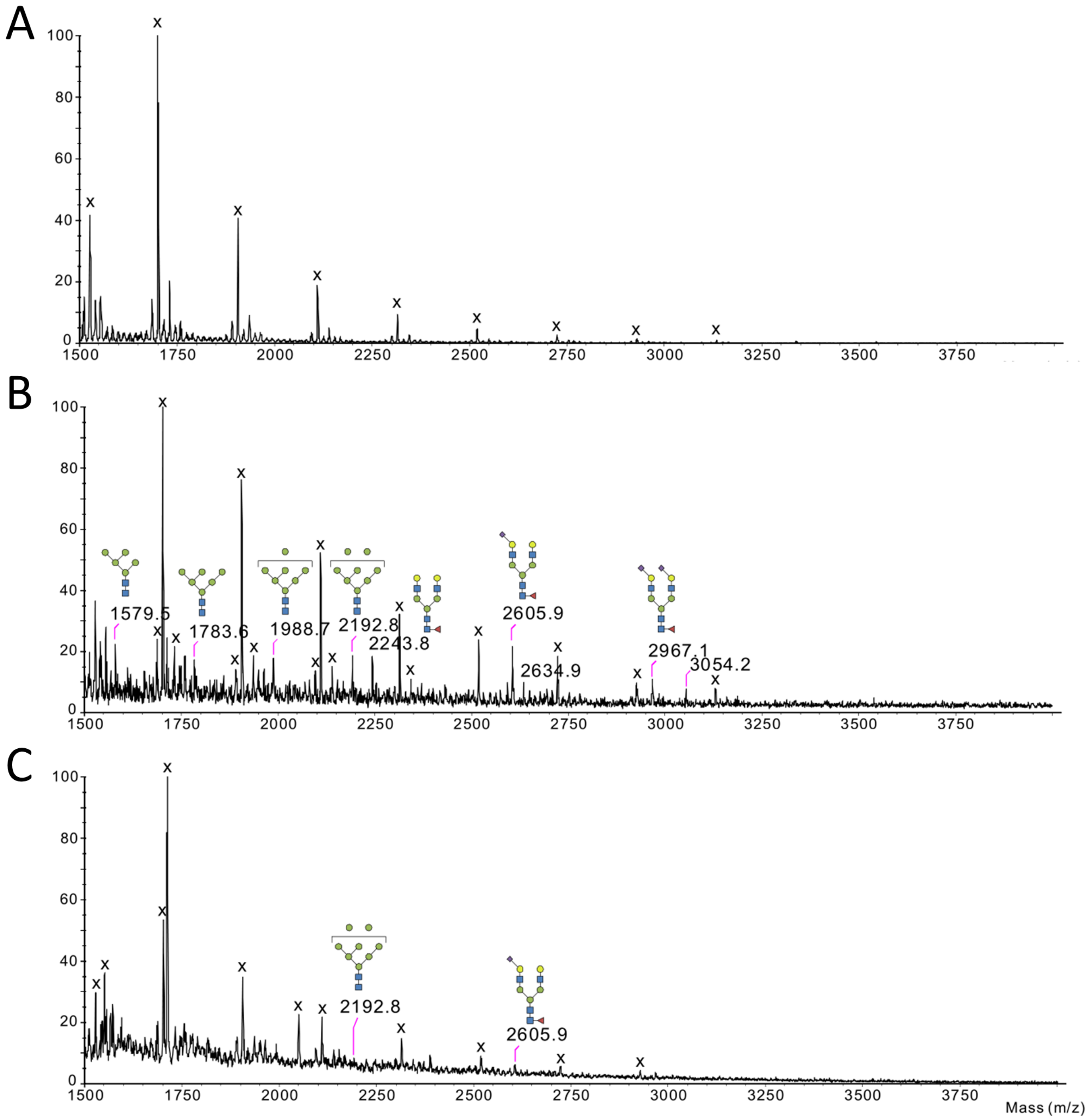

Figure S1. MALDI MS/MS analysis of vesicles of HeLa cells: untreated **(A)**, treated with UV-B as positive inducer of apoptosis **(**90 sec UV-B irradiation followed by 4 h incubation**) (B)** or FimH lectin (10 FimH-MP per cell followed by 4 h incubation) **(C)**. No glycans were detected in the membranous vesicles of untreated cells. Polyhexose contamination is indicated with “x”.

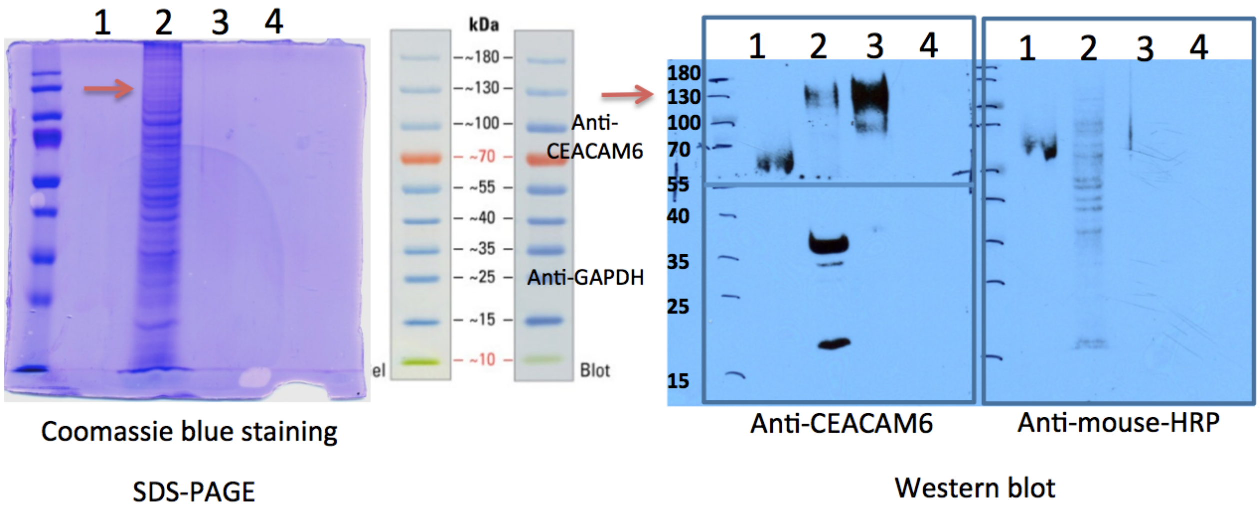

Figure S2. Coomassie blue staining and Western blot of CEACAM6 purification by immunoprecipitation:

1. Flow-through to verify antibody 9A6 (Aldevron) coupling
2. Flow-through to verify that CEACAM6 was retained by the column
3. Eluate
4. 2nd elution (to ensure complete CEACAM6 elution)

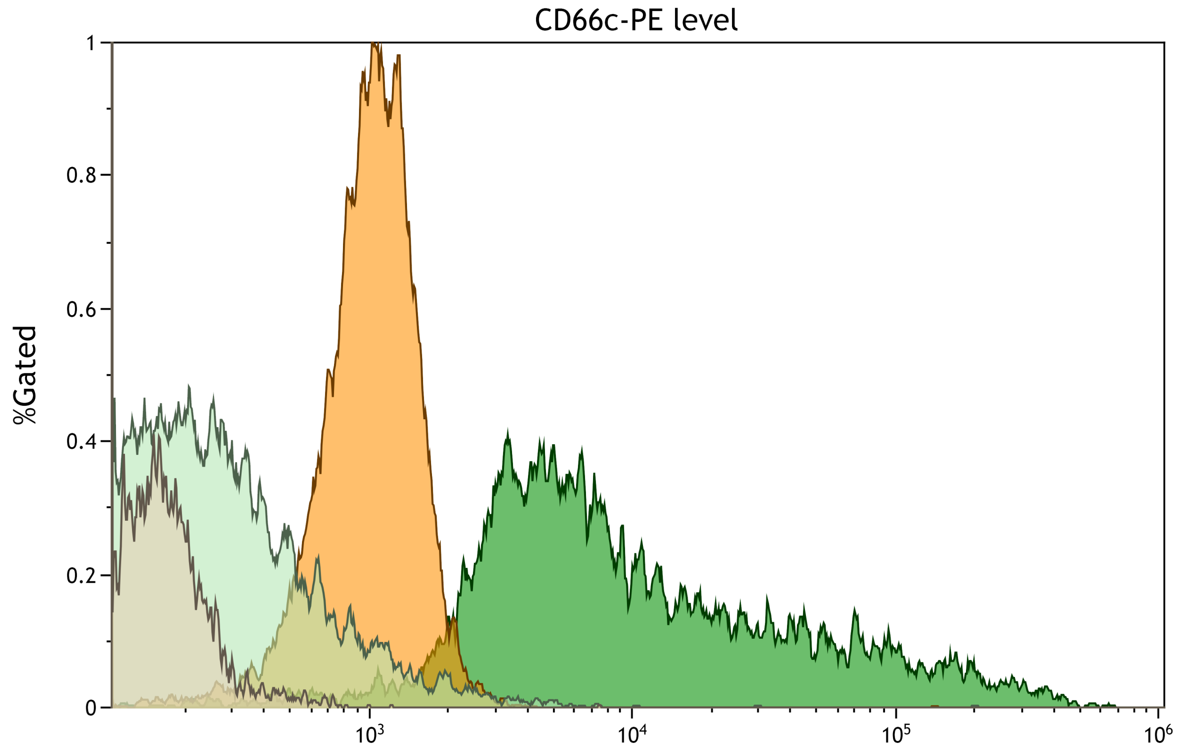

Marker	X-Med	X-GMean
HeLa unstained [cells]	105.5	122.5
Caco2 unstained [cells]	215.8	250.3
HeLa CD66c-PE [cells]	1,034.0	977.7
Caco2 CD66c-PE [cells]	7,213.8	9,864.8

Figure S3. Binding of anti-CD66c-PE antibodies (aimed to detect CEACAM6) to HeLa and Caco-2 cells:
Flow cytometric measurements with anti-CD66c-PE (anti-CEACAM6 antibody fluorescently labelled with phycoertyhrin) show an about 10-fold lower detection of CEACAM6 on HeLa *versus* Caco-2 cells.

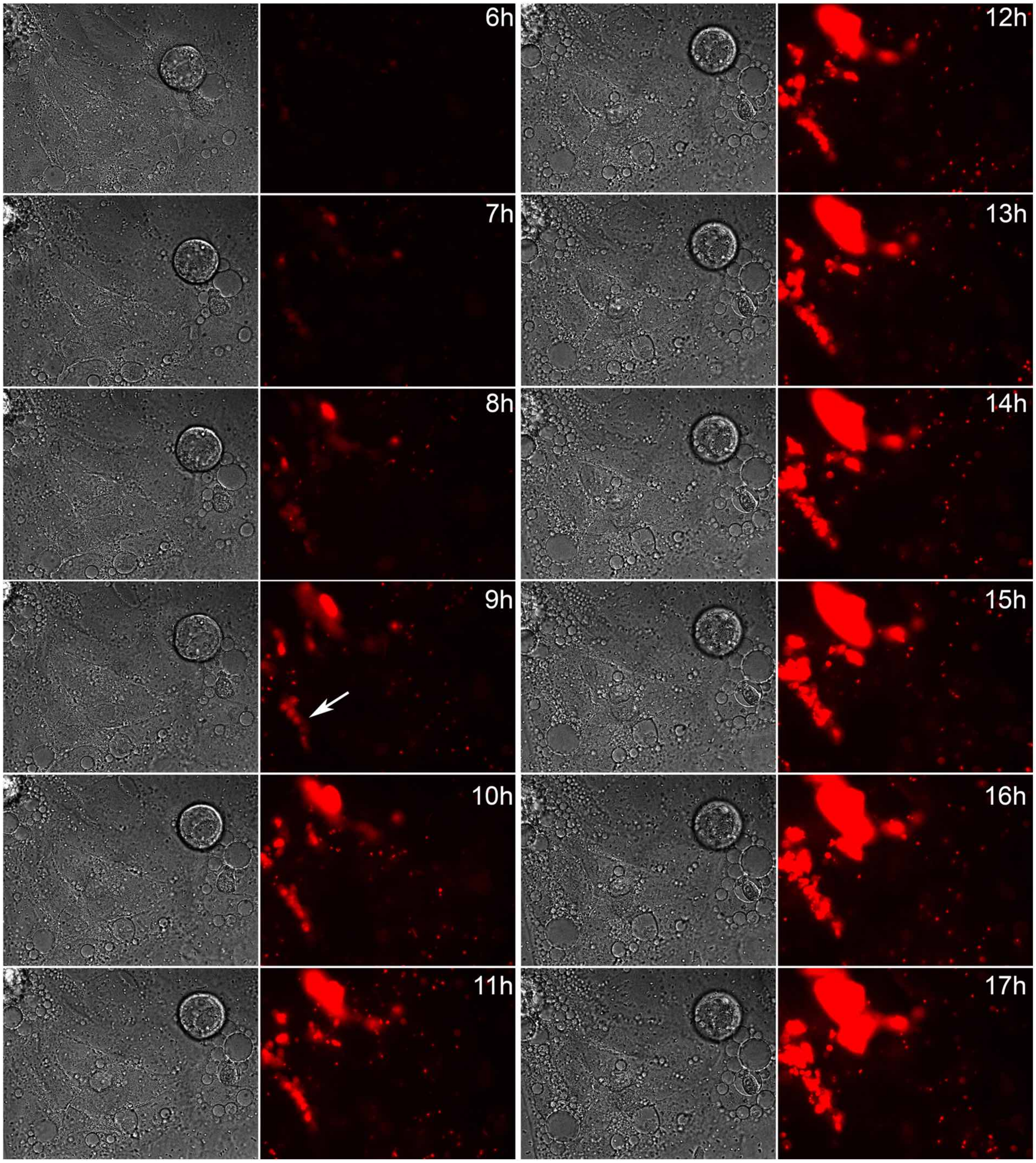

Figure S4A. Propagation of pathogenic bacteria in the intercellular spaces 6 to 17 h after infection: co-culture of the human epithelial cell line Caco-2 with the bacterial strain LF82. LF82 expresses the NIR fluorescent protein TurboFP635 (red).

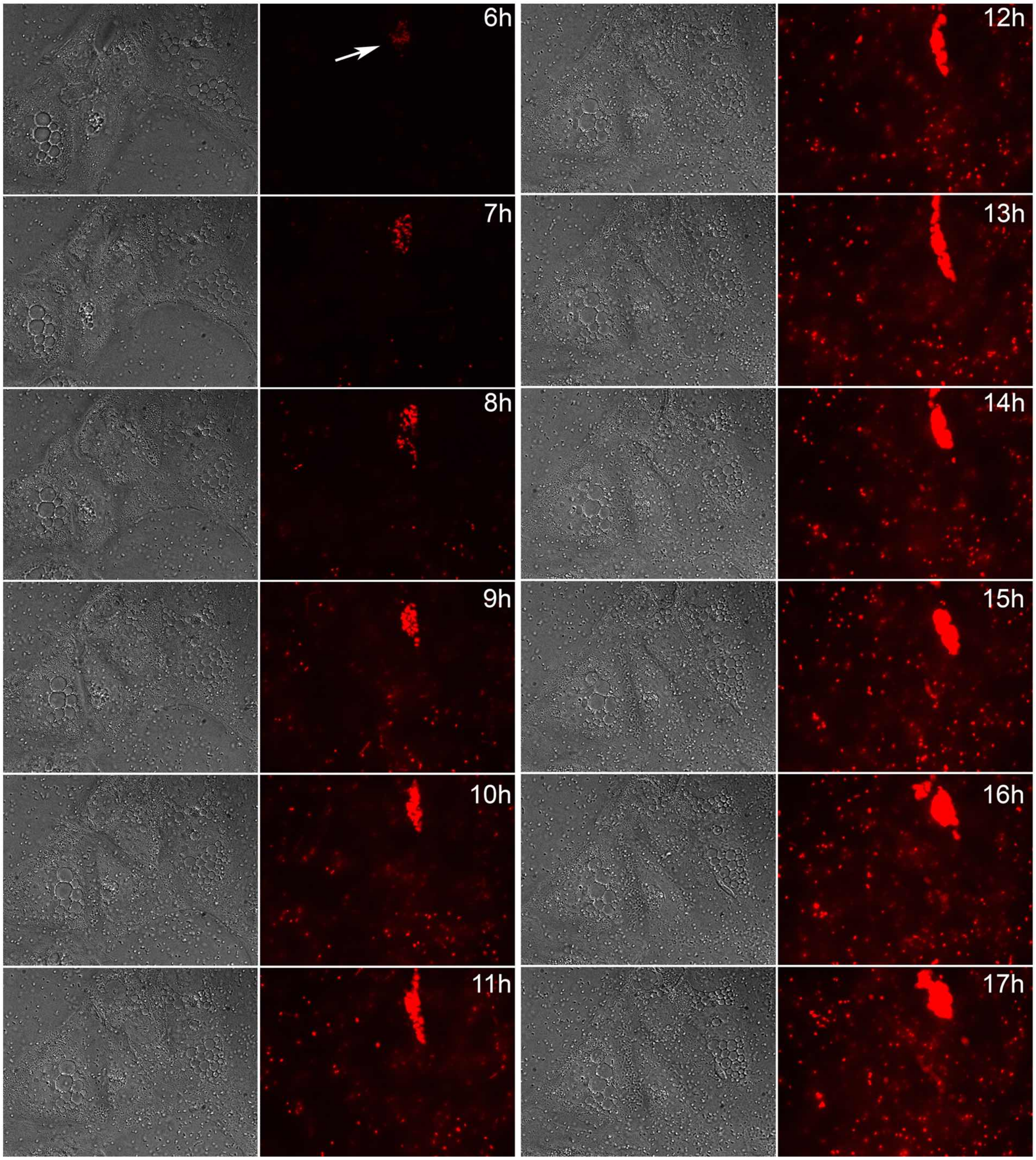

Figure S4B. The formation of intracellular bacterial communities at 6 to 17 h after initial infection: co-culture of the human epithelial cell line Caco-2 with the bacterial strain LF82. LF82 expresses the NIR fluorescent protein TurboFP635 (red).

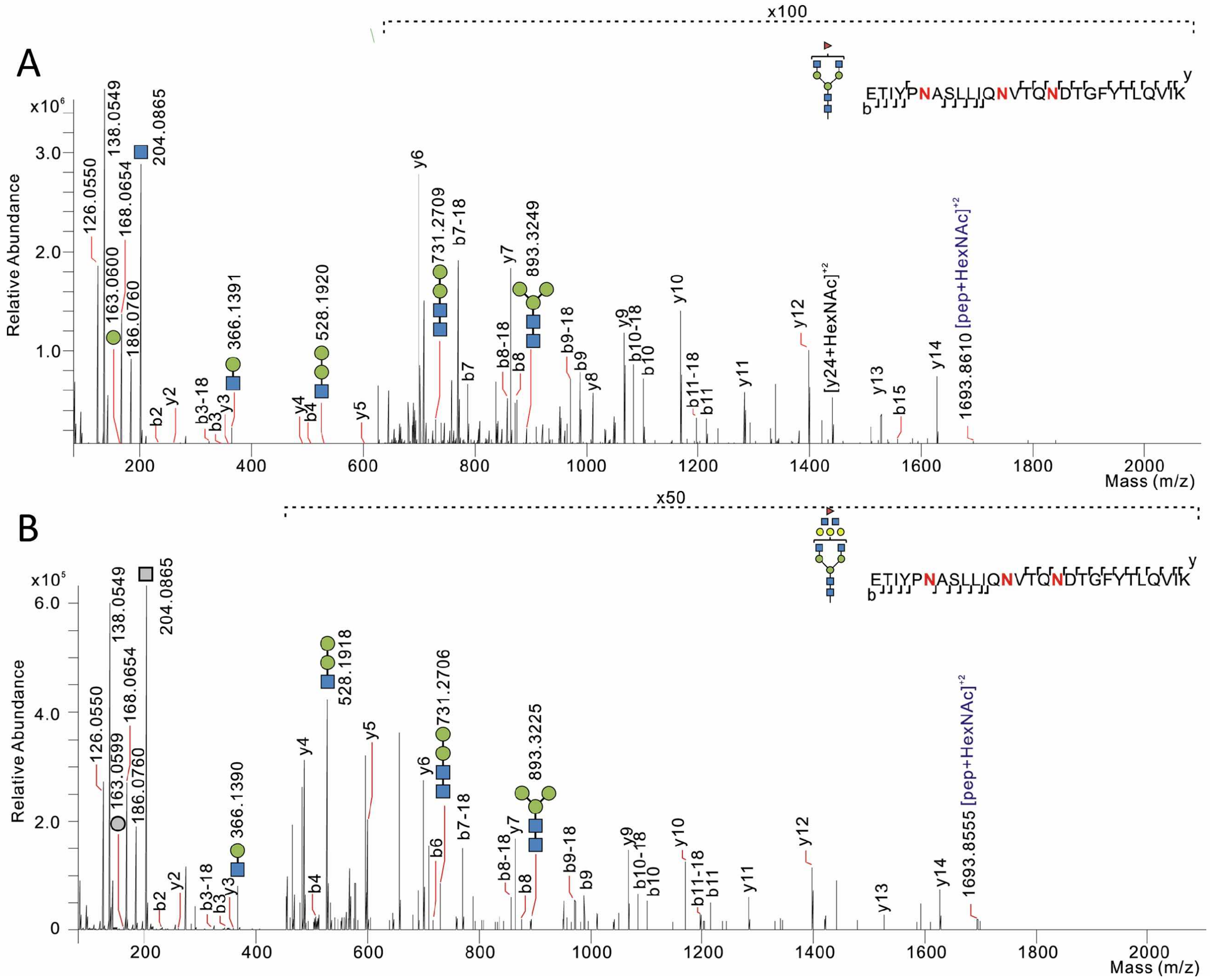

Figure S5. Glycosylation analyses of recombinant human CEACAM6 (rhCEACAM6) protein: HCD-MS/MS spectra of ETIYPNASLLIQNVTQNDTGFYTLQVIK modified with **(A)** GlcNAc4Man3Fuc1 or **(B)** GlcNAc6Man3Hex3Fuc1. The potential *N*-glycosylation sites are highlighted in bold red (positions Asn104, Asn111 and Asn115).

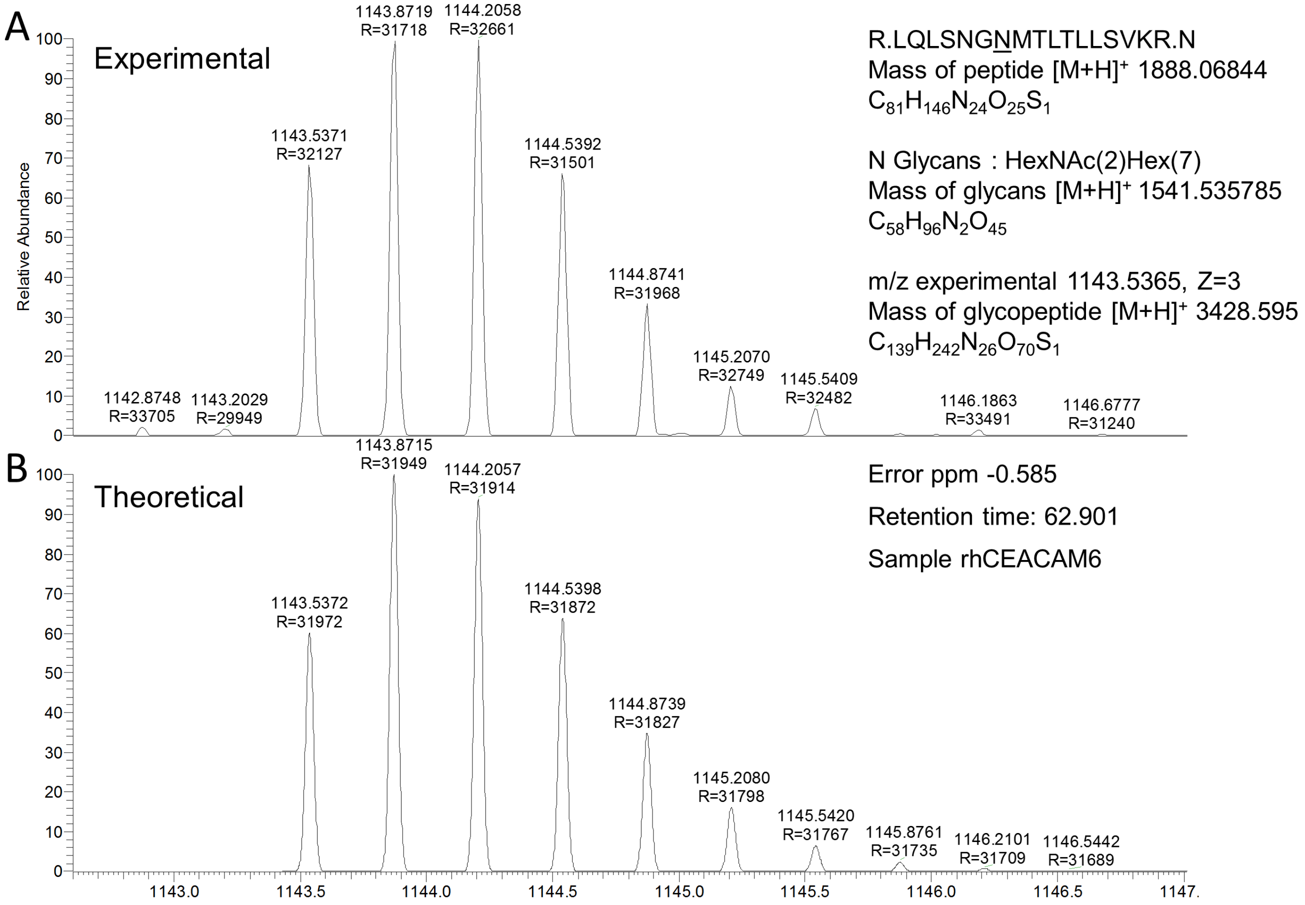

Figure S6 Isotopic profile of recombinant human rhCEACAM6 glycopeptide identified by LC-MSMS orbitrap Q-Exactive plus: **(A)** Glycopeptide at m/z 1143.5365, Z=3 identified ($[M+H]^+$ 3428.595) at retention time 62.90 min includes the amino acid sequence R.LQLSNGNMTLTLLSVKR.N ($[M+H]^+$ 1888.06844, $C_{81}H_{146}N_{24}O_{25}S_1$) and *N*-glycans HexNAc(2)Hex(7) ($[M+H]^+$ 1541.535785, $C_{58}H_{96}N_2O_{45}$). **(B)** Theoretical profile of glycopeptide at m/z 1143.53716, Z=3, $C_{139}H_{242}N_{26}O_{70}S_1$. The error is -0.585 ppm between experimental and theoretical.

1.2 Supplementary Tables

Table S1. Comparison of the highest Byonic scores in different CEACAM6 samples: rhCEACAM6 and CEACAM6 isolated from non-infected cells (control) and after infection with the bacterial strains LF82 or K12.

**Protein Name =** >sp|P40199|CEAM6_HUMAN Carcinoembryonic antigen-related cell adhesion molecule 6 OS=Homo sapiens GN=CEACAM6 PE=1 SV=3, # AAs 344								
Sample condition Name of Data	**|LogProb|**	**Best |Log Prob|**	**Best Score**	**#Spectra**	**# Unique Peptides**	**# Modified Peptides**	**% Coverage**	**Intensity**
In-Gel Control CTRL_CEACAM6	160.67	15.35	916	86	42	37	50.3	8.46E+07
In-Gel LF82 LF82_CEACAM6	196.5	13.28	935.3	105	59	54	41.6	1.16E+08
In-Gel K12 K12_CEACAM6	187.27	14.51	833.5	73	41	39	23.8	8.33E+07
eFASP recombinant rh_CEACAM6	2713.72	19.78	1375.6	1664	460	442	90.1	1.19E+10
